# Clinical Outcomes of *In Vitro* Maturation After Oocyte Retrieval With Gynecological Surgery for Refractory Polycystic Ovary Syndrome: A Retrospective Cohort Study

**DOI:** 10.3389/fendo.2022.842037

**Published:** 2022-03-04

**Authors:** Wen Zhang, Tingting Liang, Bing Han, Rui Yang, Shuo Yang, Yan Yang, Jiajia Zhang, Xiaoying Zheng, Jie Yan, Caihong Ma, Xueling Song, Jie Qiao

**Affiliations:** ^1^ Center for Reproductive Medicine, Department of Obstetrics and Gynecology, Peking University Third Hospital, Beijing, China; ^2^ National Clinical Research Center for Obstetrics and Gynecology (Peking University Third Hospital), Beijing, China; ^3^ Key Laboratory of Assisted Reproduction, Ministry of Education (Peking University), Beijing, China; ^4^ Beijing Key Laboratory of Reproductive Endocrinology and Assisted Reproductive Technology, Beijing, China; ^5^ Research Units of Comprehensive Diagnosis and Treatment of Oocyte Maturation Arrest, Chinese Academy of Medical Sciences, Beijing, China; ^6^ Department of Obstetrics and Gynecology, The Second Hospital of Shanxi Medical University, Taiyuan City, China

**Keywords:** *in vitro* maturation, IVM-surgery, polycystic ovary syndrome, FSH, LH

## Abstract

**Objective:**

To explore the clinical outcomes of unstimulated *in vitro* maturation (IVM) after oocyte retrieval with gynecological surgery (IVM-surgery) for refractory polycystic ovary syndrome (PCOS) and analyze the influencing factors.

**Methods:**

Patients with refractory PCOS who underwent unstimulated IVM-surgery from June 2014 to September 2018 were included in this retrospective cohort study. Matured IVM oocytes were freshly fertilized and subsequently frozen at the blastocyst stage. Frozen-thawed embryo transfer was then conducted according to the desire of patients. Oocytes and embryological outcomes, reproductive outcomes were evaluated. Influencing factors of oocytes and embryological outcomes were analyzed by univariate analysis and multivariate analysis. Receiver operating characteristic curves were used to evaluate the predict value of serum hormone levels for oocytes and embryological outcomes.

**Results:**

A total of 93 patients with refractory PCOS who underwent unstimulated IVM-surgery were included in this study.13 patients (13/85, 15.3%) had spontaneous pregnancy and live birth after surgery. 34 patients (34/93, 36.6%) obtained blastocysts and received embryo transfer, of which 13 patients (13/34, 38.2%) eventually achieved live birth by IVM. Higher anti-Mullerian hormone, antral follicle count and basal serum luteinizing hormone (LH) levels were strongly correlated with higher number of oocytes retrieved (*P* = 0.004, 0.004, 0.040, respectively). Higher basal serum follicle-stimulating hormone (FSH) and LH were significantly associated with higher oocyte maturation rate (*P* = 0.001 and *P* = 0.004, respectively) and blastocyst formation (*P* = 0.036 and *P* = 0.003, respectively). There was a significant linear correlation between basal serum FSH and LH (r = 0.500, *P <*0.001). What is more, basal serum FSH and LH had predictive value for oocytes and embryological outcomes.

**Conclusion:**

Unstimulated IVM-surgery provided the opportunity for both spontaneous pregnancy and assisted reproductive technology. Basal FSH and LH were significantly associated with oocyte maturation rate and blastocyst formation of unstimulated IVM-surgery.

## Introduction

Polycystic ovary syndrome (PCOS) is a highly prevalent disorder effecting reproductive-aged women worldwide, which is characterized by clinical or biochemical hyperandrogenism, ovulatory dysfunction and polycystic ovarian morphology (1). The first line treatment for PCOS is clomiphene citrate, which has an ovulation rate of 80%, but 20% of patients are still resistant to clomiphene citrate. Patients who are refractory to at least 3 clomiphene citrate treatment cycles can be diagnosed as refractory PCOS (2). Transvaginal retrieval of immature oocytes has been used for refractory PCOS patients and showed some therapeutic effects (3).

*In vitro* maturation (IVM) has been used as a technique that has led to the birth of thousands of healthy babies worldwide (4). IVM involves the retrieval of immature oocytes followed by *in vitro* culture to obtain mature oocytes, which has mainly been applied for women with PCOS to avoid the risk of ovarian hyperstimulation syndrome (5) and fertility preservation strategy for women with cancer (6). Compared to conventional *in vitro* fertilization (IVF) treatment, IVM has fewer complications due to the lower hormonal side effects, which is less costly and simpler to perform.

Currently, retrieval of immature oocytes and subsequent IVM in combination with gynecological surgery (IVM-surgery) for benign gynecological diseases is poorly studied. Oocyte retrieval for IVM does not require synchronization with the menstrual cycle or lengthy pretreatment (7), which provides a possibility to perform IVM simultaneously with gynecological surgery. For instance, some patients may undergo a hysteroscopic examination or laparoscopic surgery, transvaginal retrieval of immature oocytes during endoscopic gynecological procedures could be considered. On one hand, IVM-surgery for patients who will undergo subsequent IVF could avoid the additional procedures of ovarian stimulation and oocyte retrieval, which will reduce surgery associated complications, cost and extra time requirements. On the other hand, for patients who wish to have spontaneous pregnancy, IVM-surgery could restore normal pelvic anatomy, create conditions for spontaneous pregnancy and furthermore allow for fertility preservation (8).

Our center of reproductive medicine previously had conducted the first prospective cohort study to evaluate the effectiveness and safety of unstimulated IVM associated with laparoscopy/hysteroscopy procedures. The previous study included a total of 158 women with refractory PCOS who underwent IVM-surgery. Matured IVM oocytes obtained from these women were either freshly fertilized and subsequently frozen at the blastocyst stage (fresh oocyte group, n = 46) or the oocytes were frozen (frozen oocyte group, n = 112) for fertility preservation followed by later thawing for insemination and cleavage embryo transfer (ET) (n = 33). In the fresh oocyte group, the clinical pregnancy rate and live birth rate per ET cycle were 69.2% and 53.8%, respectively. In the frozen oocyte group, the clinical pregnancy rate and live birth rate per ET cycle were 28.6% and 19.1%, respectively. The previous study concluded that IVM-surgery on unstimulated ovaries is a novel option that can be considered for fertility preservation for women requiring gynecological surgery. However, the sample size of previous studies was small. Due to the poor fertility outcome of frozen oocyte group, we did not continue the corresponding study, but focused on the IVM population. The previous study only included 46 patients of which matured IVM oocytes were freshly fertilized and subsequently frozen at the blastocyst stage. We subsequently expanded the sample size to recruit more patients with refractory PCOS who were willing to undergo unstimulated IVM-surgery. Therefore, this study retrospectively analyzed the clinical outcomes of these patients and analyzed the influencing factors.

## Materials and Methods

### Study Design and Patients

This was a retrospective cohort study of patients with refractory PCOS who underwent laparoscopic and/or hysteroscopic surgery for benign indications at the Reproductive Centre of the Peking University Third Hospital from June 2014 to September 2018. This study was conducted based on a finished prospective cohort study (Clinical Trials ID: chictr-ONC-17011861). The study was approved by the Ethics Committee of Peking University (2014S2004).

The inclusion criteria were as follows: (1) infertile women with refractory PCOS who has undergone laparoscopy/hysteroscopy and conducted IVM during the surgery. PCOS was diagnosed according to the Rotterdam ESHRE/ASRM consensus criteria (2004); (2) age ≤ 38 years old; (3) basal serum follicle-stimulating hormone (FSH) < 10 IU/L. Exclusion criteria were: (1) with previous ovarian surgery, pelvic mass of unclear origin, bilateral ovarian cyst or clinical suspicion of endometrial hyperplasia; (2) with other endocrine severe diseases, immune diseases, and tumors. After retrieval of immature oocytes, *in vitro* culture was conducted to obtain mature oocytes and then matured IVM oocytes were freshly fertilized and subsequently frozen at the blastocyst stage. The recruited patients were followed up until March 2020.

### Transvaginal Oocyte Retrieval and Gynecological Surgery

No stimulation with gonadotropin and no human Chorionic Gonadotropin (hCG) trigger treatment before immature follicle aspiration was conducted. Immature follicle aspiration was carried out by an experienced operator before gynecological surgery. All visible follicles (2-10mm) were aspirated with 19-gauge single-lumen aspiration needles (K-OPS-7035-REH-ET; Cook, Queensland, Australia) under a suction pressure of 80 mmHg. Laparoscopic ovarian drilling was the most common surgical procedure performed during this study. Each patient received only once laparoscopic ovarian drilling. For patients with anti-mullerian hormone (AMH) < 5 ng/ml, laparoscopic ovarian drilling is not considered. As this study was conducted based on a finished prospective cohort study, detailed information can be found in XL Song’s study (8).

### IVM Culture Protocol, Oocyte Fertilization, Embryo Culture and Blastocyst Vitrification

The cumulus–oocyte complexes (COCs) were examined under a stereomicroscope (Nikon, SMZ1000, Japan), which were then transferred into IVM oocyte medium (Sage IVM media kit; Origio, Denmark) supplemented with 0.075 IU/ml of FSH and 0.075 IU/ml of luteinizing hormone (LH) (Menopur; Ferring Reproductive Health, Kiel, Germany) for maturation. Maturity of COCs was then evaluated after IVM culture for 28–32 h. Immature oocytes were further cultured for an additional 10–14 h. Only oocytes with extrusion of the first polar body were considered to be mature (metaphase II (MII) stage oocytes). Mature oocytes were inseminated by intra-cytoplasmic sperm injection, which is done routinely for IVM in our lab, but not because of male factors. All embryos were cultured in GM medium (G-M, Life Global, CT, USA) supplemented with 10% synthetic serum substitute (SSS; Irvine Scientific, Santa Ansa, CA, USA). Quality of embryos was evaluated according to the Istanbul Consensus Workshop on Embryo Assessment criteria (9). Day-3 embryos were further cultured to develop into the blastocyst for cryopreservation. Blastocysts were evaluated according to the Gardner morphological grading system.

### Frozen-Thawed Embryo Transfer

Blastocyst vitrification and thawing procedure were reported previously (10). Oral oestradiol valerate (Progynova, 6 mg, daily; Schering, Berlin, Germany) was initiated on the second day of the menstrual cycle. Progesterone withdrawal bleeding was administrated for anovulatory women to identify the menstrual cycle phase. Progesterone intravaginal gel (Crinone 8% 90 mg, daily; Merck Serono, USA) combined with oral dydrogesterone was administered for 7 days when the endometrial thickness reached 8mm. After 7 days of progesterone treatment, one blastocyst was transferred into the uterine. Detailed information can be found in XL Song’s study (8).

### Clinical Data and Definitions

The basic characteristics of the participants, such as age, body mass index (BMI), infertility type, infertility duration, insulin resistance, AMH, basal serum FSH, luteinizing hormone (LH), testosterone levels on the 3rd menstruation day, antral follicle count (AFC) and free androgen index (FAI) (total testosterone×100/sex hormone binding globulin) were evaluated. Insulin resistance was defined as index of homeostasis model assessment of insulin resistance (HOMA-IR)> 2.5. HOMA-IR = fasting blood glucose (mmol/L) × fasting insulin level (mIU/L)/22.5. Oocytes, embryological outcomes and reproductive outcomes of IVM-surgery were also evaluated. Oocyte maturation rate was defined as the number of MII oocytes divided by all the cultured oocytes. Live birth was defined as the birth of at least one living child, irrespective of the duration of gestation. Patients were considered lost to follow-up when we cannot reach them in any way.

### Statistical Analysis

Characteristics were presented as mean ± standard deviation (SD) or median (interquartile range, IQR) for continuous variables, and percentages for categorical variables. Comparisons between ratios were performed using the Chi-square test or Fisher exact test. Continuous variables were analyzed by t-test or nonparametric tests. Logistic regression models were used to estimate the effect of serum hormone levels on oocytes and embryological outcomes. Associations between two parameters were evaluated by Spearman’s test and scatter plots were drawn. Receiver operating characteristic curves (ROC) were made to determine the relationship between serum hormone levels and oocytes and embryological outcomes. Cut-off values of number of oocytes retrieved and oocyte maturation rate for predicting blastocysts formation was determined by ROC curve and its clinical practical value. Statistical significance was set at a probability (P) value < 0.05. Analysis was performed using statistical package for social science (SPSS) software, version 25.0 (IBM, Armonk, New York, USA).

## Results

### Characteristics and Oocytes Outcomes of Refractory PCOS Patients Who Underwent IVM-Surgery

A total of 93 patients with refractory PCOS who underwent unstimulated IVM-surgery were included in this study. Baseline characteristics, surgical indications as well as oocytes and embryological outcomes are presented in **Table 1**.

**Table 1:** Characteristics of the study participants who underwent IVM-surgery.

Baseline characteristics, n = 93	
Age (years), mean ± SD	28.6 ± 3.1
BMI (kg/m^2^), mean ± SD	24.8 ± 3.2
Primary infertility, %	73.1%
Infertility duration > 60 months, %	26.9%
HOMA-IR, %	
HOMA-IR > 2.5, %	51.6%
HOMA-IR ≤ 2.5, %	48.4%
FSH (mIU/ml), mean ± SD	5.6 ± 1.6
LH (mIU/ml), mean ± SD	9.2 ± 5.3
AMH (ng/ml), mean ± SD	12.7 ± 5.6
Testosterone (nmol/L), median (IQR)	1.7 (1.0, 2.3)
FAI, median (IQR)	7.1 (4.1, 13.0)
Antral follicle count, mean ± SD	19.7 ± 8.7
PCOS type, %	
with hyperandrogenism, %	61.3%
without hyperandrogenism, %	38.7%
Clomiphene citrate treatment cycles, mean ± SD	3.3 ± 0.6
**Indications for surgery, n = 93**	
LOD for clomiphene citrate resistance, n (%)	54 (58.1%)
Tubal pathology, n (%)	29 (31.2%)
Ovarian cyst, n (%) (Teratoma n = 2, endometrioma n = 2)	4 (4.3%)
Uterine pathology, n (%) (Leiomyoma n = 1, uterine septum n = 2, intrauterine adhesions n = 1, endometrial polyps n = 2)	6 (6.5%)
**Oocytes and embryological outcomes of IVM-surgery cycles, n = 93**	
Oocytes retrieved, median (IQR)	12 (8, 19)
Matured oocyte rate, median (IQR)	41.5% (27.3%, 56.0%)
Blastocyst formation, %	45.2%

IVM, in vitro maturation; BMI, body mass index.

HOMA-IR, index of homeostasis model assessment of insulin resistance.

FAI, free androgen index; LOD, laparoscopic ovarian drilling.

IQR, interquartile range; SD, standard deviation.

### Reproductive Outcomes of Patients With IVM-Surgery

**Figure 1:**
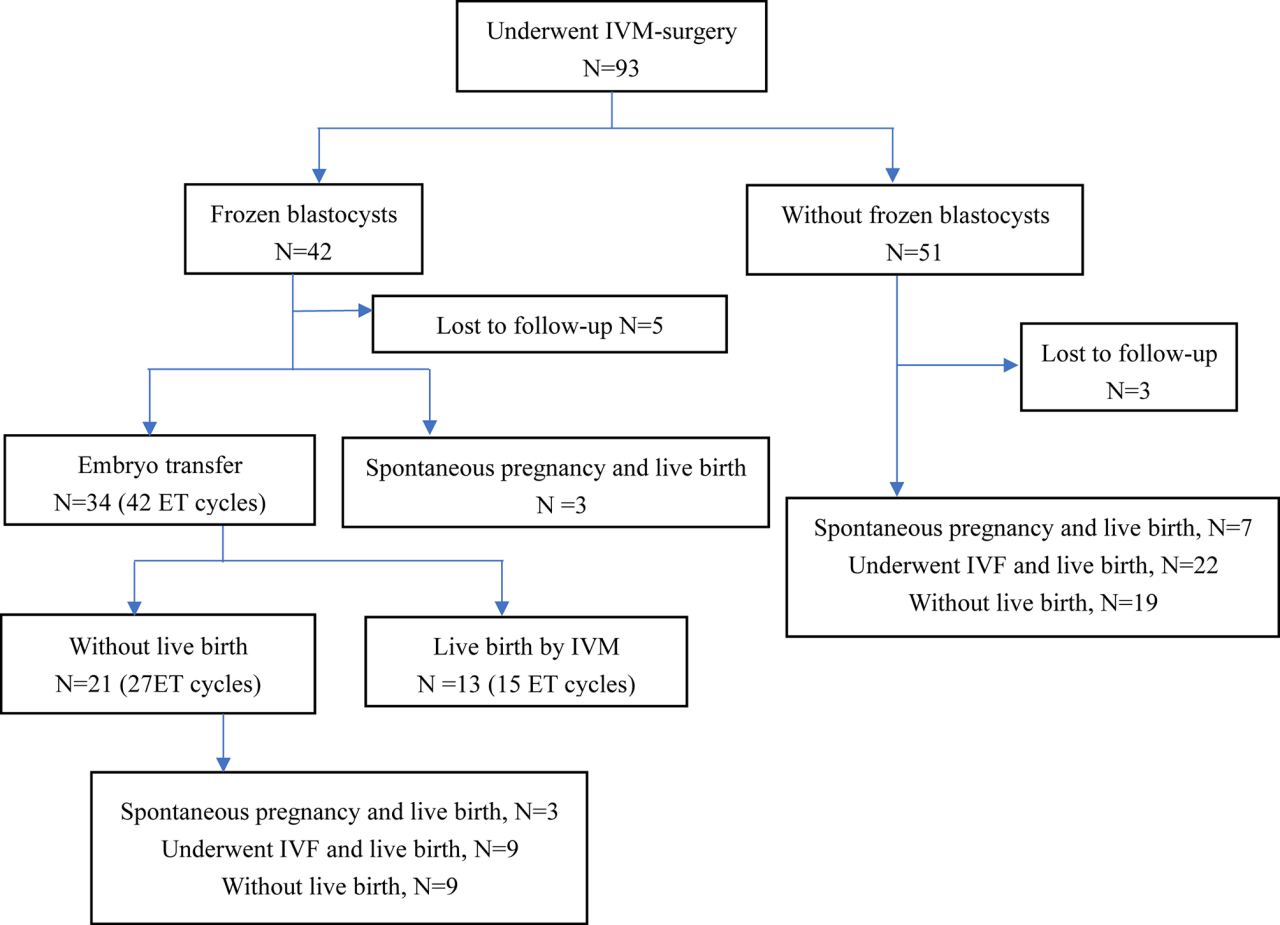
Reproductive outcomes of patients with IVM-surgery. ET, embryo transfer; IVM, *in vitro* maturation; IVF, *in vitro* fertilization.

Of the 93 patients with refractory PCOS who underwent unstimulated IVM-surgery, 8 patients (8/93, 8.6%) were lost to follow-up. Reproductive outcomes of 85 patients were shown in **Figure 1**. After IVM-surgery, 47 patients met the criteria for natural conception after surgery and attempted to be pregnant without assisted reproductive technology, of which 13 patients (13/85, 15.3%) had a live birth. On the other hand, among the 93 patients, 42 patients (42/93, 45.2%) obtained blastocysts, 34 patients received embryo transfer, and 13 patients (13/34, 38.2%) eventually achieved live birth, as shown in **Figure 1**. Due to the limited sample size, we did not explore the influencing factors of natural conception and live birth through IVM.

### Factors Influencing the Number of Oocytes Retrieved During IVM-Surgery for Refractory PCOS Patients

We divided the patients into two groups according to the number of oocytes retrieved, with a threshold of 15. There were statistically significant differences in serum AMH, AFC and basal serum LH levels between the two groups, as shown in **Table 2**. Multivariate analysis results further confirmed the correlation between serum hormone level, AFC and number of oocytes retrieved (**Table 3**). AMH, AFC and LH levels were higher in patients with number of oocytes retrieved > 15 oocytes when compared to patients with fewer oocytes (*P* = 0.004, 0.004, 0.040, respectively). However, there were no statistically significant differences in other indicators between the two groups.

**Table 2:** Factors influencing the number of oocytes retrieved during IVM-surgery.

	Number of Oocytes retrieved		P value*
	≤15 n = 61	>15 n = 32	
Age (years), mean ± SD	28.6 ± 3.0	28.6 ± 3.3	0.978
BMI (kg/m^2^), mean ± SD	24.5 ± 3.4	25.5 ± 2.6	0.160
Primary infertility, %	70.5%	78.1%	0.430
Infertility duration > 60 months, %	23.0%	34.4%	0.238
Insulin resistance, %	46.2%	60.9%	0.263
FSH (mIU/ml), mean ± SD	5.5 ± 1.5	5.8 ± 1.8	0.320
LH (mIU/ml), median (IQR)	8.7 (3.5,12.7)	9.3 (6.2, 12.9)	0.035
AMH (ng/ml), median (IQR)	10.6 (7.0,17.0)	15.9 (11.2,18.2)	0.001
Testosterone (nmol/L), median (IQR)	1.8 (0.8,2.4)	1.9 (1.4,2.3)	0.415
FAI, median (IQR)	6.5 (4.0,12.7)	7.1 (4.2,17.0)	0.538
Antral follicle count, mean ± SD	17.5 ± 7.8	23.7 ± 9.1	0.001

^*^P value of univariate analysis. IVM, in vitro maturation; BMI, body mass index; SD, standard deviation; FAI, free androgen index; IQR, interquartile range.

**Table 3:** Multivariate analysis of factors influencing oocytes and embryological outcomes.

		OR	95%CI	P value*
Oocytes retrieved				
Basal LH		2.684	1.047, 6.878	0.040
Basal AMH		5.623	1.763, 17.936	0.004
Antral follicle count		1.087	1.027, 1.151	0.004
Matured oocyte rate				
Basal FSH		8.070	2.421, 26.893	0.001
Basal LH		4.044	1.562, 10.469	0.004
Available blastocysts formation				
Basal FSH		2.765	1.068, 7.160	0.036
Basal LH		3.945	1.583, 9.833	0.003

*Adjusted by age and BMI.

### Factors Influencing Oocyte Maturation Rate During IVM-Surgery for Refractory PCOS Patients

**Figure 2:**
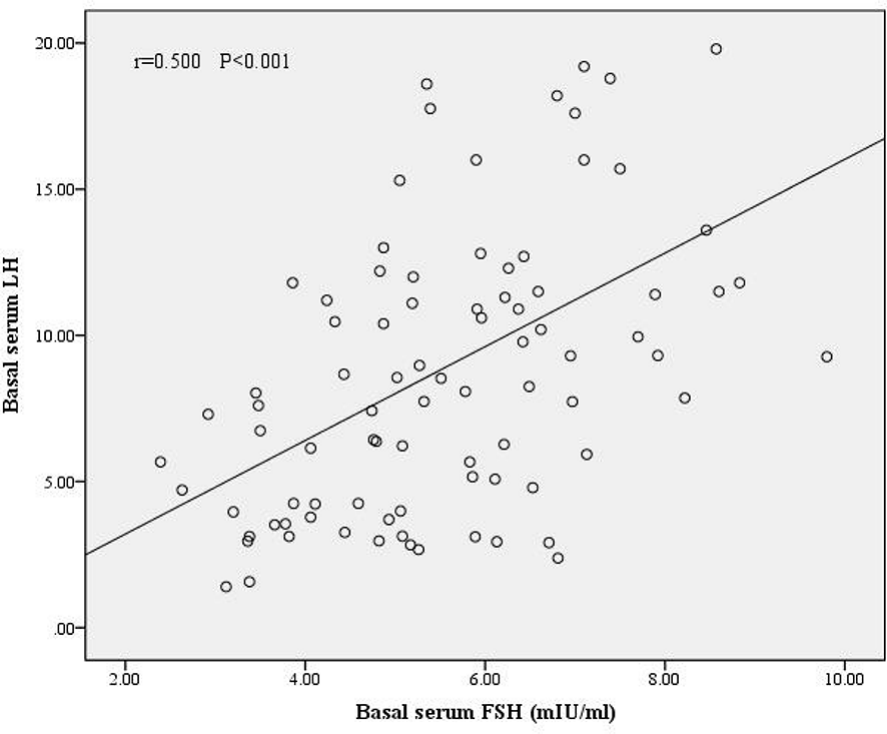
Correlation between basal serum FSH and LH. There was a significant linear correlation between FSH and LH (r = 0.500, *P* < 0.001).

**Figure 3:**
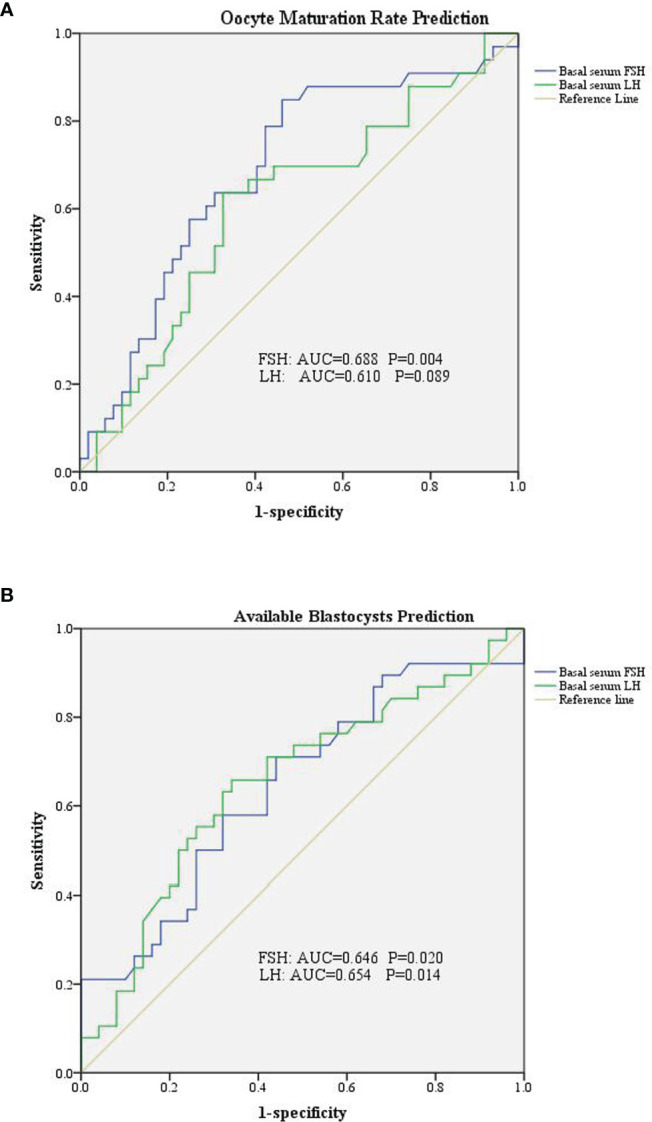
Receiver operating characteristic curve of basal serum FSH and LH in prediction of oocytes and embryological outcomes. **(A)** Basal serum FSH has predictive value for oocyte maturation rate. **(B)** Both basal serum FSH and basal serum LH has predictive value for available blastocysts.

We divided the patients into two groups according to oocyte maturation rate, with a threshold of 50%. There were statistically significant differences in basal serum FSH and basal serum LH levels between the two groups, as shown in **Table 4**. Multivariate analysis results further confirmed the above results (**Table 3**). FSH and LH levels were higher in patients with oocyte maturation rate > 50% (*P* = 0.001 and *P* = 0.004, respectively). Besides, Spearman’s test demonstrated that there was a significant linear correlation between basal serum FSH and LH (r = 0.500, *P* < 0.001) (**Figure 2**). Furthermore, the predictive value of basal serum FSH and LH on oocyte maturation rate was analyzed by ROC curve. The areas under the ROC curve (AUC) of FSH and LH were 0.688 and 0.610 (*P* = 0.004 and *P* = 0.089, respectively), respectively. Therefore, serum basal FSH showed predictive value for oocyte maturation rate (**Figure 3A**).

**Table 4:** Factors influencing oocyte maturation rate during IVM-surgery.

	Matured oocyte rate		P value*
	≤50% n = 53	>50% n = 37	
Age (years), mean ± SD	29.1 ± 3.1	28.1 ± 3.1	0.138
BMI (kg/m^2^), mean ± SD	24.8 ± 3.2	25.0 ± 3.2	0.673
Primary infertility, %	67.9%	78.4%	0.276
Infertility duration > 60 months, %	28.3%	24.3%	0.675
Insulin resistance, %	50.0%	57.7%	0.554
FSH (mIU/ml), mean ± SD	5.2 ± 1.5	6.1 ± 1.6	0.008
LH (mIU/ml), median (IQR)	7.6 (3.5,11.9)	10.4 (5.8,16.4)	0.036
AMH (ng/ml), median (IQR)	12.8 (7.4,16.8)	11.4 (8.5,19.3)	0.971
Testosterone (nmol/L), median (IQR)	1.8 (1.2,2.3)	1.9 (0.9,2.4)	0.621
FAI, median (IQR)	6.3 (3.8,12.0)	8.8 (4.2,15.1)	0.347
Antral follicle count, mean ± SD	20.3 ± 9.6	18.2 ± 7.3	0.269

^*^P value of univariate analysis; IVM, in vitro maturation; BMI, body mass index; SD, standard deviation; FAI, free androgen index; IQR, interquartile range.

### Factors Influencing Blastocysts Formation During IVM-Surgery for Refractory PCOS Patients

As for blastocysts formation, there were also statistically significant differences in basal serum FSH and basal serum LH levels between the two groups, as shown in **Table 5**. Multivariate analysis results also confirmed the above results (**Table 3**). FSH and LH levels were higher in patients with blastocysts formation (*P* = 0.036 and *P* = 0.003, respectively). Besides, AUC of FSH and LH for predicting blastocysts formation were 0.646 and 0.654, respectively, with *P* = 0.020 and *P* = 0.014, respectively. Therefore, both serum basal FSH and LH showed predictive value for blastocysts formation (**Figure 3B**).

**Table 5:** Factors influencing available blastocysts formation during IVM-surgery.

	Available blastocyst formation		P value*
	Yes n = 42	No n = 51	
Age (years), mean ± SD	28.1 ± 2.9	29.1 ± 3.2	0.127
BMI (kg/m^2^), mean ± SD	24.9 ± 3.1	24.8 ± 3.2	0.779
Primary infertility, %	76.2%	70.6%	0.544
Infertility duration > 60 months, %	28.6%	25.5%	0.739
Insulin resistance, %	50.0%	53.1%	0.806
FSH (mIU/ml), mean ± SD	6.1 ± 1.8	5.2 ± 1.4	0.012
LH (mIU/ml), median (IQR)	11.1 (7.5,15.6)	6.1 (3.5,10.9)	0.010
AMH (ng/ml), median (IQR)	14.4 (9.2,17.7)	12.5 (7.1,17.4)	0.512
Testosterone (nmol/L), median (IQR)	2.1 (1.2,2.4)	1.7 (1.0,2.5)	0.318
FAI, median (IQR)	7.1 (4.0,13.8)	6.5 (3.9,12.8)	0.799
Antral follicle count, mean ± SD	21.5 ± 9.6	18.1 ± 7.7	0.066

^*^P value of univariate analysis; IVM, in vitro maturation; BMI, body mass index; SD, standard deviation; FAI, free androgen index; IQR, interquartile range.

## Discussion

In the present study, patients with refractory PCOS undergoing unstimulated IVM-surgery were included. IVM-surgery offered the opportunity for both spontaneous pregnancy and assisted reproductive technology. Total of 26 patients (26/85, 30.6%) finally achieved live birth after IVM-surgery by spontaneous pregnancy or by IVM. For refractory PCOS patients undergoing IVM-surgery, higher AMH, AFC and LH levels were strongly correlated with higher number of oocytes retrieved. Higher basal FSH and LH were significantly associated with higher oocyte maturation rate and blastocyst formation, what is more, basal FSH and LH had predictive value for oocytes and embryological outcomes.

Our study did demonstrate the effectiveness of IVM for refractory PCOS patients, with 45.2% (42/93) of patients obtaining blastocysts, 81.0% (34/42) of which underwent embryo transfer and 38.2% (13/34) finally had live births through IVM. IVM is not appropriate for normally ovulating patients with dominant follicles, because dominant follicles can inhibit the growth of other follicles and promote atresia, leading to adverse outcomes for IVM (8). All patients included in this study had refractory PCOS, and these patients basically had no dominant follicle growth, which met IVM requirements. Song XL’s study also confirmed that for IVM-surgery, 41.3% (19/46) of patients obtaining blastocysts, 68.4% (13/19) of which underwent embryo transfer and 53.8% (7/13) finally had live births through IVM. Thus, IVM-surgery for refractory PCOS is feasible.

IVM-surgery not only provided patients with the opportunity to have a pregnancy through assisted reproductive technology, but also provided them with the opportunity to have spontaneous pregnancy. Patients included in this study were infertile patients with refractory PCOS, thus these patients would continue to be infertile and unable to obtain a spontaneous pregnancy without the intervention of IVM-surgery. With treatment of IVM-surgery, 13 patients (13/85, 15.3%) had spontaneous pregnancy and live birth after surgery, which proved that IVM-surgery provided conditions for spontaneous pregnancy. Effect of IVM-surgery on spontaneous pregnancy could be explained as follows. On one hand, the procedure itself could remove the pathological factors affecting infertility. On the other hand, the procedure of laparoscopic ovarian drilling and aspiration of transvaginal oocyte retrieval reduced the number of theca cells, which decreased the production of androgen (11). What was more, transvaginal oocyte retrieval reduced ovarian punctures and did not require electrocautery, which led to less trauma to the ovarian tissue and protected the ovarian reserve. All these created conditions for spontaneous pregnancy.

As for the IVM technology, the technique is not widely used because there are still many unresolved issues, such as the oocyte retrieval protocol, the composition of the culture medium, and the culture time, which are still uncertain. In the present study, we also explored the factors influencing the oocytes and embryological outcomes.

Basal AMH, AFC and LH showed strong correlation with the number of oocytes retrieved. AMH is specifically expressed in granulosa cells of small growing follicles, which is important indicators reflecting ovarian reserve (12). It has been proposed as a surrogate for antral follicle count in the diagnosis of PCOS. Previous studies have noted that there was a linear relationship between AMH and oocyte yield after ovarian stimulation (13, 14). Therefore, AMH is of value in predicting oocyte yield for IVM. AFC is also an important indicator of ovarian reserve, which is the number of antral follicles detected by transvaginal ultrasound before oocyte retrieval. Therefore, theoretically, the AFC is a direct parameter of the number of oocytes obtained. The present study showed that AFC was strongly correlated with the number of oocytes retrieved, which was also consistent with the results of previous studies (15, 16). LH showed positive correlations with AMH in the PCOS patients (17), which may be the reason why LH was found to correlate with the number of oocytes obtained in our study.

Basal FSH was closely related to oocyte maturation rate and blastocyst formation in present study. FSH stimulates the growth and maturation of immature oocytes into mature (Graafian) secondary follicles before ovulation (18). For PCOS patients, low levels of FSH makes it difficult to reach the “threshold” required for follicle maturation, which is an important cause of follicle arrest (19). Therefore, FSH pretreatment before IVM or addition of FSH to the culture system of IVM, theoretically, could promote follicular growth and maturation and thus improve the oocytes and embryological outcomes. In our study, median of matured oocyte rate in IVM was 41.5%, which was lower than the previous study in which Walls et al. reported that matured oocyte rate was as high as 73.0% (5). The reason for the low rate of matured oocyte rate in our study was that we did not use any gonadotrophins stimulation before transvaginal oocyte retrieval procedure, however Walls et al. administered gonadotrophins for 3–6 days before IVM (FSH priming). Anderiesz et al. demonstrated that the addition of recombinant FSH alone into the culture system increased the maturation of human oocytes compared to the maturation of oocytes with no hormones (20). Further, Jesús Cadenas noted that FSH improved oocyte nuclear maturation at concentrations above 70 IU/L suggesting a threshold for FSH during IVM of *in vitro* collected human oocytes from small antral follicles (21). Our study also found similar conclusions: compared with those with lower basal serum FSH, those with higher basal FSH had higher oocyte maturation rate and higher probability of blastocyst acquisition. Meanwhile, FSH showed certain predictive value for oocytes and embryological outcomes. These studies suggested that FSH pretreatment before transvaginal retrieval of immature oocytes or culture medium supplemented with FSH could improve IVM oocytes and embryological outcomes. Therefore, in subsequent clinical practice, we could give appropriate FSH stimulation before IVM-surgery.

Basal LH also showed close relationship with oocyte maturation rate and blastocyst formation. Patients with PCOS often have increased pulsatile release of pituitary gonadotropins, as evidenced by abnormally elevated LH (22). LH has a role in promoting ovulation and oocyte maturation in humans. Child TJ reported a maturation rate of 80.3% for IVM outcomes with natural cycle in patients with PCOS (23), which was much higher than the data in our study (41.5%), which may be due to the fact that Child TJ’s study used a modified natural cycle protocol for IVM using hCG to induce ovulation 3-5 days prior to ovulation retrieval, whereas all patients in our study did not have hCG trigger therapy prior to ovulation retrieval. However, recent evidence suggested that IVM culture systems plus LH were not associated with oocyte maturation. C. Accardo et al. demonstrated that LH alone had no effect on the developmental of potential of bovine oocytes (24), which neither improved matured oocyte rate nor blastocyst formation, however recombinant FSH alone or in combination with recombinant LH may have stimulatory effects on the progression of the meiotic cycle (24). It can be explained by the phenomenon that cumulus and granulosa cells of medium-sized (2–6 mm) follicles express FSH receptors but could hardly express LH receptors (25). Rubens Fadini et al. noted that hCG could promote in vivo meiotic resumption and progression to the MII stage in oocytes of follicles of a diameter of 10–12 mm (26). Therefore, LH could hardly affect the growth and development of small follicles. In this study, the follicles obtained from patients with refractory PCOS were all small follicles, so theoretically, basal serum LH did not affect the development of follicles. However, our study found that LH was significantly correlated with oocyte maturation rate and blastocysts formation, which may be related to several reasons. On one hand, this study showed the significant linear correlation between LH and FSH in included patients, therefore due to the linear correlation between FSH and LH, the effect of FSH on oocyte maturation and blastocysts formation could lead to the significant correlation between LH and oocyte maturation and blastocysts formation. In other words, although theoretically basal serum LH did not affect the development of small follicles as we have discussed above, the direct impact of FSH on follicles and linear correlation between LH and FSH led to the phenomenon that LH was significantly correlated with oocyte maturation rate and blastocysts formation. One the other hand, another possible reason was that the level of LH represented the severity of PCOS, which itself affected follicular growth and development through other pathways, and therefore showed a significant correlation between LH and oocyte maturation rate and blastocysts formation. And whether LH could affect follicular growth and development through other unexplored ways needs to be confirmed by further studies.

Both FSH and LH play important roles in follicular growth, development and maturation. Previous studies have also demonstrated that FSH combined with LH can improve follicle and embryo outcomes for IVM. C Accardo compared the effects of four different IVM culture medium on sheep oocytes (IVM culture medium with recombinant FSH (r-FSH) alone, recombinant LH (r-LH) alone, r-FSH and r-LH simultaneously and without gonadotropin), which discovered the highest maturation rate was reached in the r-FSH/r-LH group (91.9%) (24). Anderiesz et al. demonstrated that although r-FSH combination with r-LH did not significantly increase matured oocyte rate, it could improve human embryonic developmental competence (20). Fadini R assessed and compared the clinical efficiency of four different priming approaches for normo-ovulatory women: no priming, hCG (10,000 IU), FSH (150 IU/d for 3 days from day 3), and FSH/hCG (26). They found that FSH/hCG priming generated the highest amount of MII oocytes and clinical pregnancy rates were much higher in the FSH/hCG treated women compared with all the other treatments. We speculated that FSH combined with LH can affect follicles of different stages and sizes. FSH promotes follicle growth and LH promotes maturation of grown follicles, thus jointly improves oocytes and embryological outcomes. Therefore, for refractory PCOS patients, appropriate FSH stimulation before IVM-surgery can be applied without LH stimulation simultaneously because of the absence of LH receptor in small follicles from refractory PCOS patients, however, whether LH should be added after FSH stimulation depends on the size and stage of follicles.

There were some advantages and disadvantages of this study. To the best of our knowledge, this study was the largest to date in exploring unstimulated IVM-surgery. The study confirmed the feasibility of unstimulated IVM-surgery. More importantly, the clinical practice significance of IVM-surgery is that it opens up new options for assisted reproduction, which is a novel option that can be considered for fertility preservation for women requiring gynecological surgery, without affecting the effect of surgery. After the IVM-surgery, the patient has both the chance of spontaneous pregnancy and the choice of assisted reproduction, and when the patient chooses assisted reproduction after IVM-surgery, they already have had a transplantable blastocyst. What is more, this study found the factors affecting oocytes and embryological outcomes. These significant findings suggested that appropriate gonadotropins stimulation could be given before IVM-surgery in subsequent assisted reproductive clinical practices. The disadvantage of this study was that due to the limited sample size, the influencing factors of live birth in IVM-surgery could not be further explored. Subsequent studies could further explore the influencing factors of live birth in IVM-surgery and the effectiveness of gonadotropins stimulation before IVM-surgery.

## Conclusion

Unstimulated IVM-surgery provided the opportunity for both spontaneous pregnancy and assisted reproductive technology. Basal FSH and basal LH were significantly associated with oocyte maturation rate and blastocyst formation of unstimulated IVM-surgery.

## Data Availability Statement

The raw data supporting the conclusions of this article will be made available by the authors, without undue reservation.

## Ethics Statement

The studies involving human participants were reviewed and approved by Ethics Committee of Peking University. Written informed consent for participation was not required for this study in accordance with the national legislation and the institutional requirements.

## Author Contributions

CM, XS, JY, and JQ contributed to conception and design of the study. RY, SY, YY, JZ, XZ, JY, and XS were involved in data acquisition. WZ, TL, and BH organized the database. WZ and TL performed the statistical analysis. WZ wrote the first draft of the manuscript. TL and BH wrote sections of the manuscript. All authors contributed to the article and approved the submitted version.

## Funding

This study was supported by the National Natural Science Foundation of China (No.81521002), the major consulting research project of the Chinese Academy of Engineering (No. 2020-XZ-22), and the CAMS Innovation Fund for Medical Sciences (2019-I2M-5-001).

## Conflict of Interest

The authors declare that the research was conducted in the absence of any commercial or financial relationships that could be construed as a potential conflict of interest.

## Publisher’s Note

All claims expressed in this article are solely those of the authors and do not necessarily represent those of their affiliated organizations, or those of the publisher, the editors and the reviewers. Any product that may be evaluated in this article, or claim that may be made by its manufacturer, is not guaranteed or endorsed by the publisher.

## References

[1] Lizneva D Suturina L Walker W Brakta S Gavrilova-Jordan L Azziz R. Criteria, Prevalence, and Phenotypes of Polycystic Ovary Syndrome. Fertil Steril (2016) 106(1):6–15. doi: 10.1016/j.fertnstert.2016.05.003 27233760

[2] Ghomian N Khosravi A Mousavifar N. A Randomized Clinical Trial on Comparing the Cycle Characteristics of Two Different Initiation Days of Letrozole Treatment in Clomiphene Citrate Resistant PCOS Patients in IUI Cycles. Int J Fertil Steril (2015) 9(1):17–26. doi: 10.22074/ijfs.2015 25918588 PMC4410033

[3] Chen ZJ Li Y Zhao LX Jiang JJ Tang R Sheng Y. Treatment of Polycystic Ovarian Syndrome With Anovulatory Infertility by Ultrasound-Guided Immature Follicle Aspiration. Zhonghua fu chan ke za zhi (2005) 40(5):295–8. doi: 10.3760/j.issn:0529-567x.2005.05.003 15938775

[4] Chian RC Xu CL Huang JY Ata B. Obstetric Outcomes and Congenital Abnormalities in Infants Conceived With Oocytes Matured In Vitro. Facts Views Vision ObGyn (2014) 6(1):15–8. PMC4085998 25009721

[5] Walls ML Hunter T Ryan JP Keelan JA Nathan E Hart RJ. *In Vitro* Maturation as an Alternative to Standard *In Vitro* Fertilization for Patients Diagnosed With Polycystic Ovaries: A Comparative Analysis of Fresh, Frozen and Cumulative Cycle Outcomes. Hum Reprod (Oxford England) (2015) 30(1):88–96. doi: 10.1093/humrep/deu248 25355587

[6] Berwanger AL Finet A El Hachem H le Parco S Hesters L Grynberg M. New Trends in Female Fertility Preservation: *In Vitro* Maturation of Oocytes. Future Oncol (London England) (2012) 8(12):1567–73. doi: 10.2217/fon.12.144 23231518

[7] Creux H Monnier P Son WY Tulandi T Buckett W. Immature Oocyte Retrieval and In Vitro Oocyte Maturation at Different Phases of the Menstrual Cycle in Women With Cancer Who Require Urgent Gonadotoxic Treatment. Fertil Steril (2017) 107(1):198–204. doi: 10.1016/j.fertnstert.2016.09.041 27810160

[8] Song XL Lu CL Zheng XY Nisenblat V Zhen XM Yang R. Enhancing the Scope of *In Vitro* Maturation for Fertility Preservation: Transvaginal Retrieval of Immature Oocytes During Endoscopic Gynaecological Procedures. Hum Reprod (Oxford England) (2020) 35(4):837–46. doi: 10.1093/humrep/dez273 32154563

[9] Balaban B Brison D Calderon G Catt J Conaghan J Cowan L. The Istanbul Consensus Workshop on Embryo Assessment: Proceedings of an Expert Meeting. Hum Reprod (Oxford England) (2011) 26(6):1270–83. doi: 10.1093/humrep/der037 21502182

[10] Zheng X Wang L Zhen X Lian Y Liu P Qiao J. Effect of Hcg Priming on Embryonic Development of Immature Oocytes Collected From Unstimulated Women With Polycystic Ovarian Syndrome. Reprod Biol Endocrinol: RB&E (2012) 10:40. doi: 10.1186/1477-7827-10-40 22621829 PMC3499152

[11] Fernandez H Morin-Surruca M Torre A Faivre E Deffieux X Gervaise A. Ovarian Drilling for Surgical Treatment of Polycystic Ovarian Syndrome: A Comprehensive Review. Reprod Biomed Online (2011) 22(6):556–68. doi: 10.1016/j.rbmo.2011.03.013 21511534

[12] Dewailly D Andersen CY Balen A Broekmans F Dilaver N Fanchin R. The Physiology and Clinical Utility of Anti-Mullerian Hormone in Women. Hum Reprod Update (2014) 20(3):370–85. doi: 10.1093/humupd/dmt062 24430863

[13] Nelson SM Yates RW Fleming R. Serum Anti-Müllerian Hormone and FSH: Prediction of Live Birth and Extremes of Response in Stimulated Cycles–Implications for Individualization of Therapy. Hum Reprod (Oxford England) (2007) 22(9):2414–21. doi: 10.1093/humrep/dem204 17636277

[14] La Marca A Sighinolfi G Radi D Argento C Baraldi E Artenisio AC. Anti-Mullerian Hormone (AMH) as a Predictive Marker in Assisted Reproductive Technology (ART). Hum Reprod Update (2010) 16(2):113–30. doi: 10.1093/humupd/dmp036 19793843

[15] Filippi F Martinelli F Paffoni A Reschini M Raspagliesi F Somigliana E. Fertility Preservation in Women With Malignancies: The Accuracy of Antral Follicle Count Collected Randomly During the Menstrual Cycle in Predicting the Number of Oocytes Retrieved. J Assist Reprod Genet (2019) 36: (3):569–78. doi: 10.1007/s10815-018-1377-0 PMC6439113 30478807

[16] Guzman L Ortega-Hrepich C Polyzos NP Anckaert E Verheyen G Coucke W. A Prediction Model to Select PCOS Patients Suitable for IVM Treatment Based on Anti-Mullerian Hormone and Antral Follicle Count. Hum Reprod (Oxford England) (2013) 28(5):1261–6. doi: 10.1093/humrep/det034 23427238

[17] Cui Y Shi Y Cui L Han T Gao X Chen ZJ. Age-Specific Serum Antimüllerian Hormone Levels in Women With and Without Polycystic Ovary Syndrome. Fertil Steril (2014) 102(1):230–6.e2. doi: 10.1016/j.fertnstert.2014.03.032 24746743

[18] Holesh JE Bass AN Lord M. Physiology, Ovulation. StatPearls. Treasure Island (FL: StatPearls Publishing Copyright © 2021, StatPearls Publishing LLC (2021). 28723025

[19] Coyle C Campbell RE. Pathological Pulses in PCOS. Mol Cell Endocrinol (2019) 498:110561. doi: 10.1016/j.mce.2019.110561 31461666

[20] Anderiesz C Ferraretti A Magli C Fiorentino A Fortini D Gianaroli L. Effect of Recombinant Human Gonadotrophins on Human, Bovine and Murine Oocyte Meiosis, Fertilization and Embryonic Development In Vitro. Hum Reprod (Oxford England) (2000) 15(5):1140–8. doi: 10.1093/humrep/15.5.1140 10783367

[21] Cadenas J Nikiforov D Pors SE Zuniga LA Wakimoto Y Ghezelayagh Z. A Threshold Concentration of FSH Is Needed During IVM of Ex Vivo Collected Human Oocytes. J Assist Reprod Genet (2021) 38(6):1341–8. doi: 10.1007/s10815-021-02244-8 PMC8266958 34050448

[22] Baskind NE Balen AH. Hypothalamic-Pituitary, Ovarian and Adrenal Contributions to Polycystic Ovary Syndrome. Best Pract Res Clin Obstet Gynaecol (2016) 37:80–97. doi: 10.1016/j.bpobgyn.2016.03.005 27137106

[23] Child TJ Abdul-Jalil AK Gulekli B Tan SL. *In Vitro* Maturation and Fertilization of Oocytes From Unstimulated Normal Ovaries, Polycystic Ovaries, and Women With Polycystic Ovary Syndrome. Fertil Steril (2001) 76(5):936–42. doi: 10.1016/S0015-0282(01)02853-9 11704114

[24] Accardo C Dattena M Pilichi S Mara L Chessa B Cappai P. Effect of Recombinant Human FSH and LH on In Vitro Maturation of Sheep Oocytes; Embryo Development and Viability. Anim Reprod Sci (2004) 81(1-2):77–86. doi: 10.1016/j.anireprosci.2003.10.004 14749050

[25] van Tol HT van Eijk MJ Mummery CL van den Hurk R Bevers MM. Influence of FSH and Hcg on the Resumption of Meiosis of Bovine Oocytes Surrounded by Cumulus Cells Connected to Membrana Granulosa. Mol Reprod Dev (1996) 45(2):218–24. doi: 10.1002/(SICI)1098-2795(199610)45:2<218::AID-MRD15>3.0.CO;2-X 8914080

[26] Fadini R Mignini Renzini M Dal Canto M Epis A Crippa M Caliari I. Oocyte In Vitro Maturation in Normo-Ovulatory Women. Fertil Steril (2013) 99(5):1162–9. doi: 10.1016/j.fertnstert.2013.01.138 23433517

